# Targeted delivery of immuno-RNase may improve cancer therapy

**DOI:** 10.1186/s12935-018-0546-7

**Published:** 2018-04-16

**Authors:** Miaonan Sun, Liankun Sun, Dejun Sun, Chunmei Zhang, Mei Li

**Affiliations:** 1 Department of Pathophysiology, College of Basic Medical Sciences, Jilin University, Changchun, 130021 China; 2 Department of Biomedicine, Regeneration Medicine Institute, College of Pharmacy, Jilin University, Changchun, 130021 China

**Keywords:** Onconase, Immunotoxin, Apoptosis, Target therapy, Anticancer

## Abstract

**Background:**

Immunotoxins are typical therapeutic drugs that can target cancer cells. They exploit the affinity of specific monoclonal antibodies or ligands to cancer cells to deliver a conjugated protein toxin to target sites, thus, attacking the cancer cells.

**Methods:**

The immuno-RNase, Onc-V3, showed the stability of Onc-V3 in the blood stream. Flow cytometry showed that apoptosis occurred in the HO-8910PM cells when treated with Onc-V3. Under the confocal microscope, the green fluorescent, FITC-Onc-V3, were located in the cytoplasm, suggesting that Onc-V3 had a function in the cytoplasm of cancer cells. Moreover, after staining by DAPI, the blue fluorescent nuclei showed shrinkage and grainy. Wound healing assay showed that high concentrations of Onc-V3 inhibited cell migration and the transwell invasion assay showed that Onc-V3 could inhibit cell invasion to the basement membrane. Western blot results showed significantly decreased PARP, procaspase-9, and procaspase-3 in Onc-V3-induced apoptosis.

**Results:**

These results of the experiments in vitro had shown that the Onc-V3 could be delivered to the cancer cells accurately and it had strong cytotoxicity on high metastatic cancer cells.

**Conclusion:**

The specific toxicity of Onc-V3 on highly metastatic cancer cells can make it a promising anti-cancer drug by using V3 to target delivery of Onconase.

## Background

Chemotherapy drugs and radiotherapy have significantly increased the survival rates of tumor-bearing patients and have prolonged their lifetime [1–3]. However, these traditional cancer therapies are based on the rule that cancer cells grow faster than the normal cells thus causing the death of epithelial and hematopoietic stem cells. Therefore, tumor targeting cytotoxic agents present a prospective field of anti-cancer research [4, 5].

Immunotoxins consist of a cell-targeting domain and a cytotoxic domain, which are connected directly or through a suitable linker [6, 7]. The linker is designed to maintain the integrity of the spatial structure and biological activity of each domain.

Onconase is an immunotoxin with a molecular weight of 12 kDa and consisting 104 amino acids [8, 9]. It was purified from the embryos of the Northern leopard frog (*Rana pipiens*), and belongs to the bovine pancreatic ribonuclease A (RNase A) superfamily [8]. Onconase has specific structural features with the presence of a Cys87–Cys104 disulfide bond at the C-terminus and a pyroglutamic acid residue (Gln) at the N-terminus [10–12]. While a methionine was present in the natural Onconase as the first amino acid, the genetically engineered version lacks the first methionine and has only l% the activity of native Onconase. Stability of the Onconase tertiary structure is maintained by three disulfide bonds, which makes it resistance to hydrolysis by proteases and heat. In vivo and in vitro studies have shown that Onconase is cytotoxic to malignant mesothelioma and other malignant tumors including breast, pancreatic, and non-small cell lung cancers. Onconase also showed synergistic effect with many other chemical, synthetic, and cytokines used as anti-cancer drugs [13–17].

Recombinant Onconase has progressed to phase III clinical trials in patients with unresectable mesothelioma, and has become the first RNase to be assessed as an anti-tumor drug. In long-term clinical applications, Onconase presented the following advantages: less drug resistance, lower allergenic, lower immunogenic, and fewer side effects [13, 14]. However, the poor capacity of Onconase to target most tumor cells has limited further clinical development.

Most tumor types overexpress a cell surface receptor termed, chemokine receptor (CXCR), which is an integral membrane protein that specifically binds and responds to cytokines of the CXC chemokine family [18–20]. It represents a large family of G protein-linked receptors that contain seven transmembrane proteins. There are seven CXC chemokine receptors in mammals, named CXCR1–CXCR7.

CXCR4 (fusin) serves as the receptor for a chemokine known as SDF-1 (or CXCL12) [20, 21], and is used by HIV-1 to gain entry into target cells [22, 23]. This receptor is widely distributed in cells and is expressed on most immature and mature hematopoietic cell types. Besides, CXCR4 is also overexpressed in more than 20 types of cancers, including melanoma, glioma, acute myelogenous leukemia, chronic lymphocytic leukemia, breast, prostate, renal, pancreatic, ovarian, cervical, colon, and small-cell lung cancers [18]. Since V3 was the core sequence of SDF-1 it had a potential use in drug design to target cancer cells.

In our previous study, we constructed a tumor-activated conjugate ONC-V3 and expressed it in the yeast [24], *Pichia pastoris*. Optimizing growth and induction conditions resulted in ONC-V3 expression reaching a maximum of 160 mg/L with a purity of 95% [24]. ONC-V3 had a low hemolytic activity, which made intravenous drug delivery possible. It showed low cytotoxicity on HEK-293 cells and strong cytotoxicity on some cancer cells.

In this study, we investigated the cytotoxicity of ONC-V3 on tumor cells and explored the likely mechanism by which ONC-V3 killed cancer cells by using DNA ladder degradation, fluorescence microscopic analysis, flow cytometry and transmission electron microscopy.

## Materials and methods

### Cell lines and cell culture

The cells used in our study are as follows:

1. HEK293, a human normal embryonic kidney cell.
2. MCF-7, a human breast carcinoma cell line.
3. A549, a human lung carcinoma cell line.
4. HO-8910PM, a human ovarian neoplasm cell line.
5. Hela, a human cervical carcinoma cell line.
6. HepG2, a human liver carcinoma cell line.

All cell lines were stored at − 80 °C in our laboratory in DMEM (Invitrogen Inc.) supplemented with 20% fetal bovine serum (FBS) and 10% DMSO. Cells were cultured in DMEM with 10% FBS, 100 unit/mL penicillin, and 100 μg/mL streptomycin at 37 °C with 5% CO_2_.

### Onc-v3

ONC-V3 was expressed and purified as described in our previous study [24]. The culture was collected after 72 h, and yeast cells were removed by centrifugation at 8000 rpm for 30 min at 4 °C. The supernatant was centrifuged in an ultracentrifuge and concentrated by ultrafiltration using the Amfore Ultrafiltration Membrane 10,000. The concentrated recombinant immunotoxin was loaded onto a SP Sepharose Fast Flow column (5.0 × 20 cm) pre-equilibrated with buffer A (20 mM PB, pH 7.0). The bound recombinant immunotoxin was eluted with buffer B (1 mol/L NaCl). The eluted protein was mixed with buffer C (20 mM PB, pH 7.0) and further loaded onto the Sephadex G-75 column (1.6 × 100 cm) pre-equilibrated with the same buffer and purified protein was collected.

### Hemolysis assay of ONC-V3

ONC-V3 was incubated with sheep red cells at the final concentrations of 20, 40, 60, 80, 100, 120, 140, 160, 180, and 200 μmol/L. Erythrocytes were added to a final concentration of 1% (v/v). The components were gently mixed and incubated for 60 min at 37 °C, and centrifuged. The optical density (OD) of the supernatants was measured at 545 nm. Erythrocytes suspended in PBS served as the control for zero hemolysis (blank) and erythrocytes in 1% Triton X-100 served as the control for 100% hemolysis. Data are presented as mean ± SD of three independent experiments.

### Cytotoxicity of ONC-V3 on cell lines

The different cell lines (MCF-7, A549, HO-8910PM, Hela, HepG2, HEK293) were seeded on 96-well plates at the density of 10^4^ cells per well, and incubated at 37 °C and 5% CO_2_ for 24 h. Then, serial dilutions of ONC-V3 (0, 0.05, 0.1, 0.2, 0.4, 0.8, and 1.6 μmol/L) were added to the cells after removing the medium. Six wells were maintained for each dose. The blank control contained the dilution medium without ONC-V3. Then, the cells were incubated at 37 °C with 5% CO_2_ for 72 h. Then, the supernatants were removed and the cells were washed with PBS (pH 7.4). The cells were further incubated in medium containing 20 μL MTT solution (5 mg/mL) at 37 °C for 4 h. Then, 100 μL of dimethyl sulfoxide (DMSO) was added after removing the supernatant. OD_490_ was measured using an ELISA microplate reader. Cytotoxicity of the cancer cells was determined based on IC_50_.

### Flow cytometry measurement of apoptosis

After treating 1 × 10^6^ cells with serial dilutions of ONC-V3 (0, 0.05, 0.1, 0.2, 0.4, 0.8, 1.6 μmol/L and 3.20 μmol/L) for 48 h, the cells were collected and centrifuged at 1000 rpm for 5 min. Then, cells were washed twice with cold PBS before adding 1 mL buffer to resuspend the cells in each tube. Then, 5 μL of Annexin V and 5 μL of PI were added to each tube. After mixing thoroughly, the tubes were incubated at room temperature for 10 min and the stained cells were immediately analyzed by flow cytometry, and the excitation and emission wavelengths were 535 and 617 nm.

### DAPI staining to show apoptosis

HO-8910PM cells (1 × 10^6^) were cultured on round coverslips in 35 mm petri dishes containing DMEM for 24 h. Then, the medium was replaced with fresh culture medium containing ONC-V3 (0.4 µmol/L). After 0 and 48 h cells were washed with PBS and fixed in ice-cold methanol for 10 min, followed by an additional wash with PBS. The cells were then stained with 1 µg/mL DAPI for 15 min, and washed with PBS for 5 min. All slides were finally observed through a fluorescence microscope.

### DNA ladder

1 × 10^7^ HO-8910PM cells were collected after culture for 48 h in the medium containing serial dilutions (0.4 μmol/L) of ONC-V3. The apoptosis positive control group received 10 μmol/L cis-platinum in addition. The cells were washed three times with PBS, and then resuspended in 350 mL of lysis solution (50 mmol Tris–HCl, 0.1 mol EDTA, 0.5% SDS) and 3.5 μL (50 μg/mL) Proteinase K and incubated in a water bath at 50 °C for 2 h. Then, equal volume of phenol–chloroform–isoamyl alcohol (25:24:1) mixture was added and mixed. The resulting mixture was centrifuged at 10,000 rpm for 10 min at 4 °C. After discarding the supernatant, 1/10 volumes of 5 mol/L NaCl and 2.5 volumes of ethanol were added. The mixture was centrifuged at 12,000 rpm for 10 min at 4 °C and the pellet was washed with 75% ethanol and dried at room temperature. The pellet was re-suspended in 100 μL of TE buffer (20 mM Tris–HCl, 1 mM EDTA, pH 8.0) and incubated with 2 μL (10 mg/mL) RNase for 1 h at 65 °C and finally tested on a 1% agarose gel by electrophoresis.

### Location of polypeptides in cells

HO-8910PM cells (1 × 10^6^) were cultured in 35 mm petri dishes with round coverslip on the bottom for 24 h. The medium was replaced with fresh culture medium containing ONC-V3 (0.4 µmol/L) for 1 and 24 h. Then, the cells were stained with the mitochondria dye, Mito Tracker Red CMXRos according to the manufacturer’s protocol. After staining, cells were washed with fresh, pre-warmed growth medium three times and the cells were incubated at 37 °C for 15 min in pre-warmed growth medium containing 4% methanol. Then, cells were rinsed three times with PBS, and the slide covers were placed onto the slides and observed through a laser scanning confocal microscope.

### Wound healing assay

For wound healing assays, HO-8910PM cells (1 × 10^6^) were cultured in 6-well plates for 24 h to obtain a confluent monolayer. Vertical artificial scratches were made in the wells by scraping the cell monolayer along the diameter of each well using a 10 μL pipette tip. Cells that separated from the monolayer were removed. Then, medium was added and cells were cultured for 12 h with PBS as solvent control, or ONC-V3 (0, 0.05, 0.1, 0.2, 0.4, 0.8, 1.6 μmol/L and 3.20 μmol/L). After 24 and 48 h in culture, the microscopic images of the scratched area before and after treatment were obtained and observed through a microscope at a magnification of 100×.

### Transwell invasion assay

The effect of Onconase on HO-8910PM cell invasion was measured using a transwell assay, and conducted in 6-well plates containing microporous 8 μmol/L membranes (Corning Costar, USA). The transwell membrane was coated with matrigel (BD Biosciences, USA), which was rehydrated by adding 45 μL of serum-free medium and incubated for 4 h. Cells were incubated with serial dilutions of ONC-V3 (0, 0.2, 0.4, 0.8, and 1.6 μmol/L) for 12 h, and then 10^5^ cells were plated in the top chambers. Medium with serum was then loaded in the lower chambers. After incubation for 24 h at 37 °C with 5% CO_2_, cells on the top surface of the membrane were removed. The cells on the lower surface were fixed in 10% paraformaldehyde for 30 min and stained with hematoxylin–eosin (H&E). Invading cells from 3 random fields were counted through a light microscope at a magnification of 200×.

### Western blotting

Cells were cocultured with ONC-V3 (0.4 µmol/L) for 48 h and harvested. All cells were lysed with lysis buffer [20 mmol/L Tris–Cl (pH 8.0), 137 mmol/L NaCl, 20 mmol/L DTT, 1% NP-40, 2 mmol/L sodium vanadate, 100 µg/mL phenylmethylsulfonyl fluoride, 1 µg/mL aprotinin; 10 µL/10^6^ cells] for 40 min and the protein concentration was measured by using a BCA protein assay kit. Then, 20 μg total protein was separated on SDS-PAGE, and transferred to a PVDF membrane (Bio-Rad, USA) for western blotting. After blocking with BSA the membrane was incubated overnight at 4 °C with antibodies against PARP (1:500, ab181020, Abcam), cleaved PARP (1:500, ab32072, Abcam), pro-caspase 9 (1:500, ab76424, Abcam), pro-caspase 3 (1:500, ab134175, Abcam), caspase-3 (1:500, 9694, CST), or caspase-9 (1:500, 7237, CST). The membrane was washed and further incubated with the corresponding secondary antibodies (1:2000) conjugated to horseradish peroxidase. SuperSignal ECL (Applygen Technologic, Peking, China) was used to visualize antibody binding.

## Results

### Hemolysis of ONC-V3

**Fig. 1:**
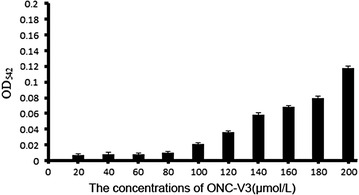
The OD_542_ of ONC-V3 interacts with red blood cells

To verify the cytotoxicity of ONC-V3, an erythrocyte hemolysis assay was performed. After incubating cells with ONC-V3 at 37 °C for 1 h, the OD_542_ was detected (Fig. 1). ONC-V3 was found to exhibit minimal hemolysis at 100 μmol/L. This concentration is 200 times more than the IC_50_ concentration (0.2–0.4 μmol/L) estimated in cytotoxicity assays. This indicates that Onconase coupled in ONC-V3 may be safely delivered in great amounts intravenously and released after selectively recognizing tumor cells.

### ONC-V3 inhibited the proliferation of tumor cells but not HEK293 cells

**Fig. 2:**
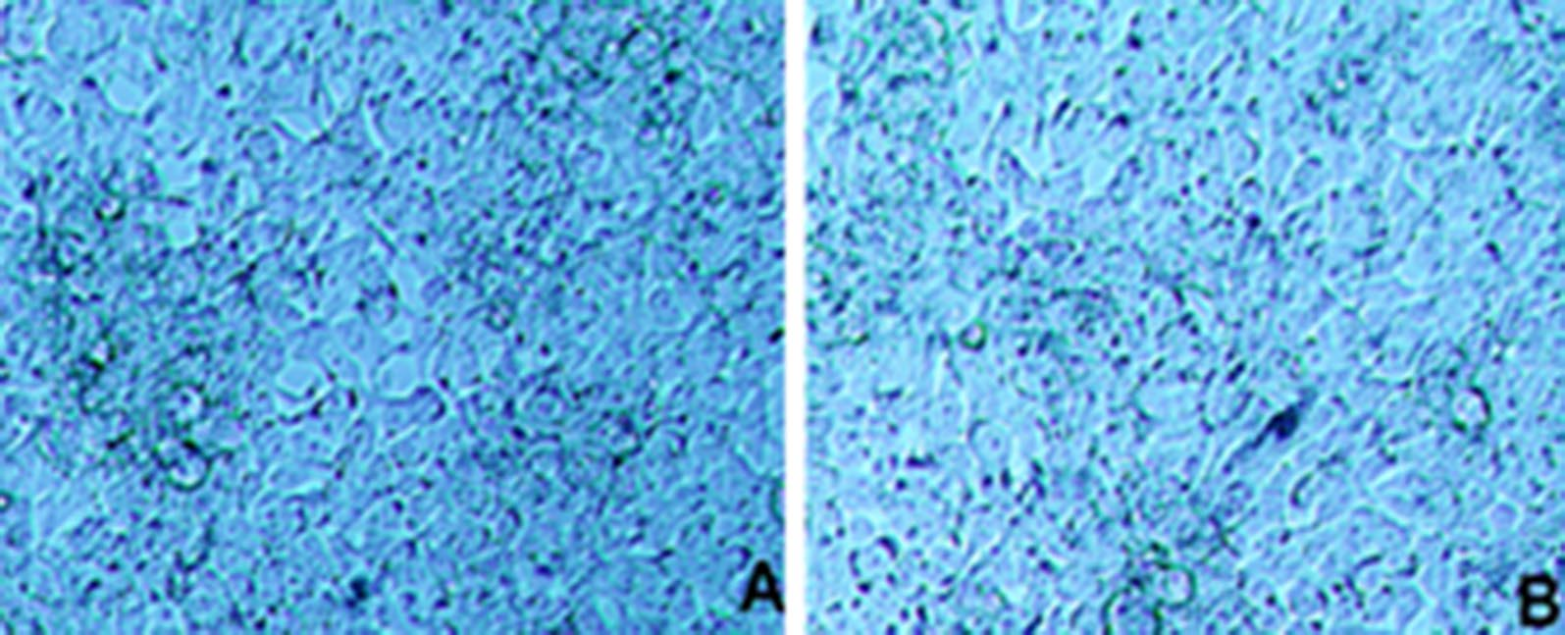
HEK293 cells treated with natural Onconase and ONC-V3. **A** The natural Onconase, **B** ONC-V3

We expressed natural Onconase and immuno-RNase, ONC-V3, to confirm their cytotoxic effects on cancer cells. Incubating ONC-V3 with multiple cell types for 48 h showed the differential effects of OVC-V3. The OD_490_ values are shown in Table 1. ONC-V3 effectively inhibited MCF-7, A549, HO-8910PM, Hela, and HepG2 cells at IC_50_ concentrations ranging from 0.19 to 0.38 μmol/L (Table 1). While both natural Onconase and immuno-RNase ONC-V3 inhibited the growth of selected tumor cell lines (Table 1). ONC-V3 did not inhibit the growth of HEK293 cell line (Fig. 2).

**Table 1:** IC_50_ of various tumor cells treated with Onc-V3

Cell lines	IC_50_ of ONC-V3 (μmol/L)	IC_50_ of Onconase (μmol/L)
MCF-7	0.36	0.83
A549	0.35	0.71
HO-8910PM	0.38	0.82
Hela	0.19	0.57
Hep G2	0.30	0.74

### ONC-V3 induced HO-8910PM cell apoptosis

**Fig. 3:**
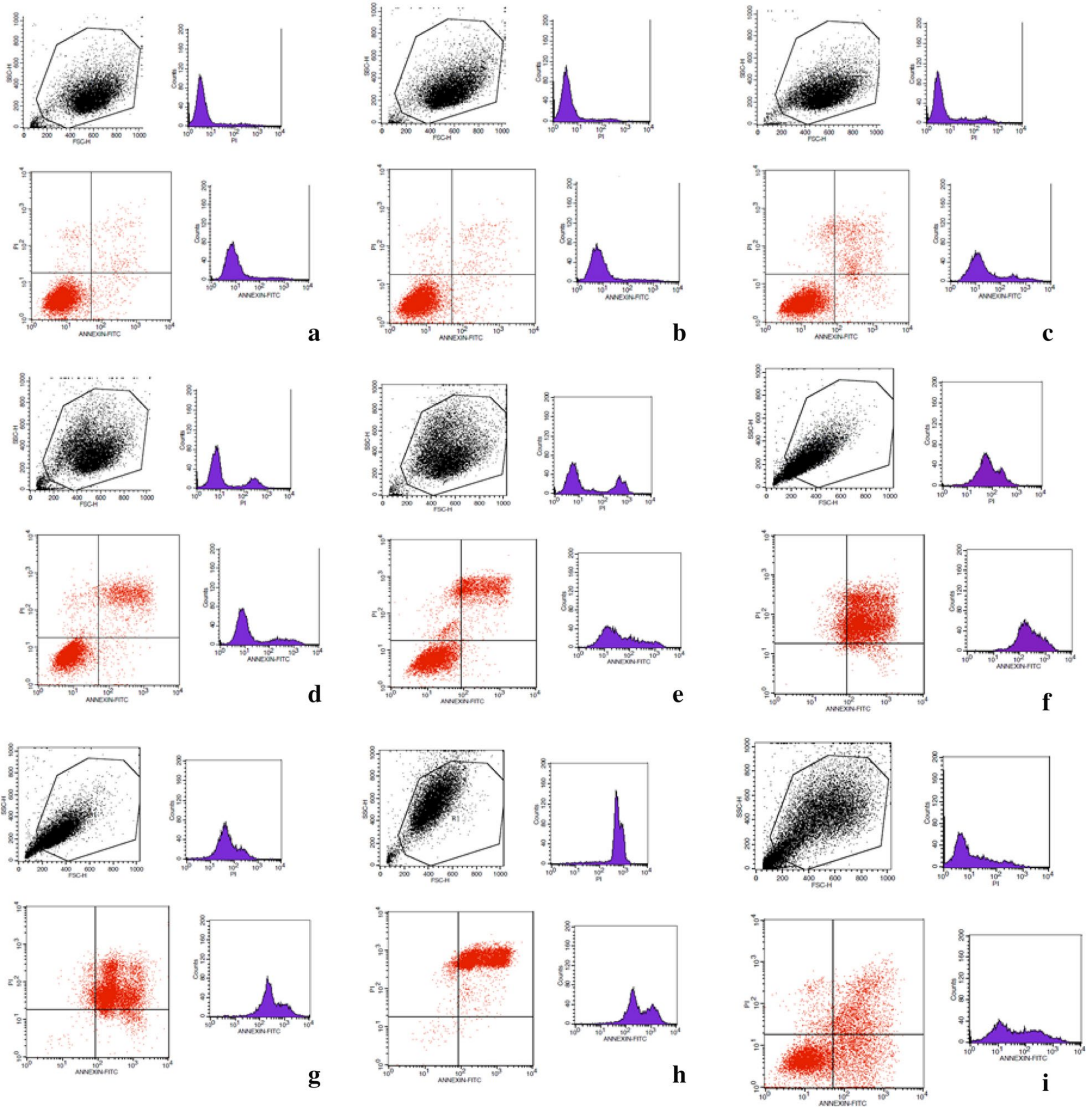
HO-8910PM cells were cultured in 0.4 µmol/L ONC-V3 for 48 h. **a** Blank control, **b**–**h** 0.05, 0.10, 0.20, 0.40, 0.80, 1.60, 3.20 μmol/L ONC-V3, **i** 5-FU. The apoptotic cells increased along with the dose. This indicated that ONC-V3 could induce apoptosis in the cancer cells

HO-8910PM cell apoptosis was then measured by flow cytometry. The cells appeared in the Q2 section, implying the occurrence of late apoptosis. Moreover, the percentage of the apoptotic cells increased in the ONC-V3 dose-dependent manner (Fig. 3).

### ONC-V3 induced HO-8910PM cells to exhibit apoptotic morphology

**Fig. 4:**
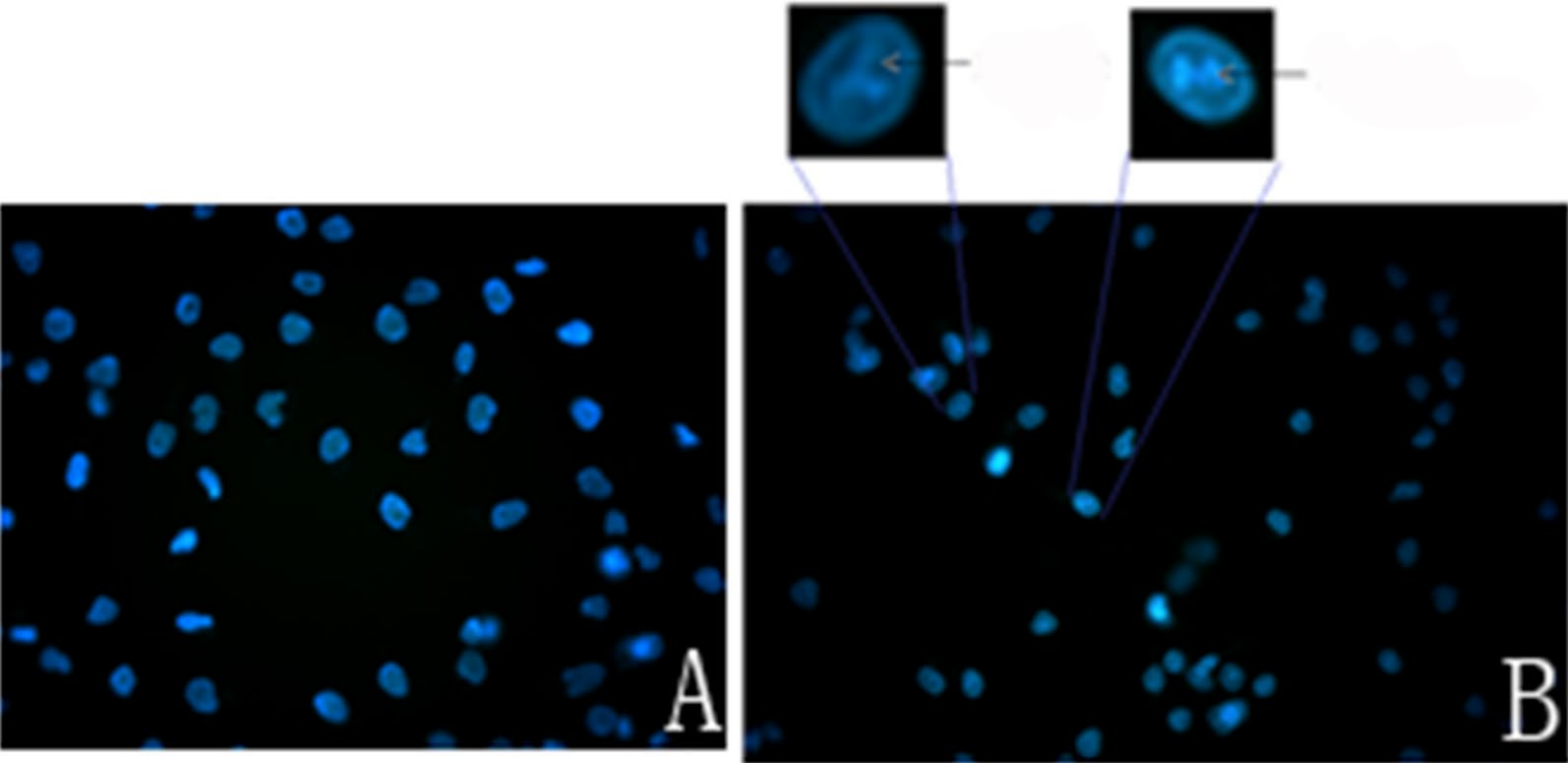
HO-8910PM cells were stained with 4,6-diamidino-2-phenylindole dihydrochloride (DAPI) after culture in 0.4 µmol/L ONC-V3 for 48 h. **A** The blank control, **B** HO-8910PM cells treated with 0.4 µmol/L ONC-V3. The nuclear chromatin condensed, aggregated, and decreased in volume. Some of the nuclear chromatin in the tumor cells was linear, indicating that the cells were apoptotic

HO-8910PM cells stained with 4,6-diamidino-2-phenylindole dihydrochloride (DAPI) revealed an apoptotic morphology after culture in 0.4 µmol/L ONC-V3 for 48 h (Fig. 4).

### ONC-V3 induced apoptosis of tumor cells

**Fig. 5:**
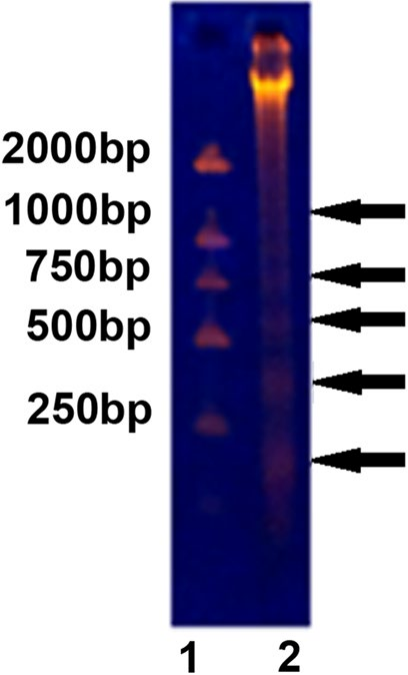
Cytotoxicity to HO-8910PM cells by gel electrophoresis fluorescence of DNA after 48 h. Lane 1: Marker; Lane 2: HO-8910PM cells with 0.4 μmol/L ONC-V3. The cells treated with high doses of the ONC-V3 and Melittin appear in a ladder pattern

HO-8910PM cells (1 × 10^7^) were collected after 48 h culture in medium containing 0.4 μmol/L ONC-V3. Then, DNA was extracted and analyzed by gel electrophoresis. The cells revealed a ladder pattern (Fig. 5) indicating the apoptosis appeared.

### ONC-V3 was distributed mainly in the cytoplasm

**Fig. 6:**
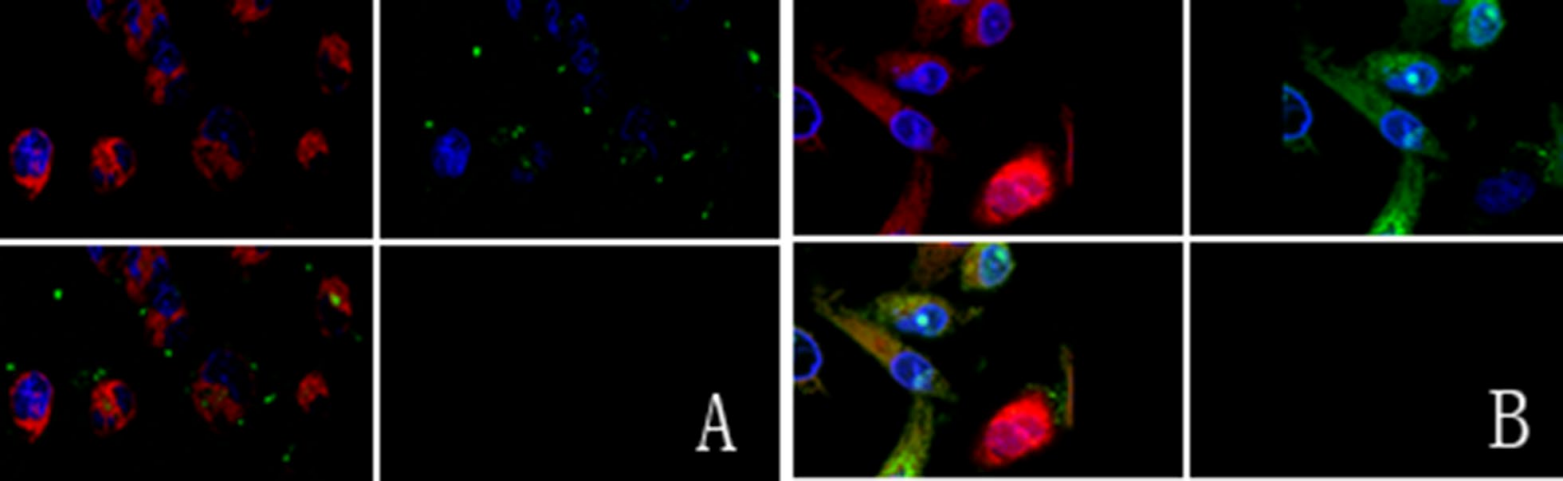
The mitochondria in the cytoplasm were stained with MitoTracker^®^ Red CMXRos red dye in the blank control group. The nucleus was stained with Hochest 33342 blue, and the red mitochondria were located in the cytoplasm surrounding the blue nucleus. **A** HO-8910PM cells were cultured in Onc-V3-containing medium for 1 h. The mitochondria were shown in red and the nucleus was in blue. The green Onc-V3 protein was located on the surface of the cell. This indicated that Onc-V3 molecules can accurately target tumor cells, and a small amount of it was in the cytoplasm. **B** HO-8910PM cells were cultured with Onc-V3 for 48 h. The green ONC-V3 was located in the cytoplasm together with the red mitochondria. This indicated that Onc-V3 is taken up by the tumor cells and plays a role in the induction of tumor cell apoptosis

ONC-V3 was labeled with fluorescein isothiocyanate (FITC) to trace its distribution in cells. When the treated cells were observed under confocal microscopy after 1 h of incubation, the immuno-RNase was found around the cytoplasmic membrane. With 48 h of incubation, it was located in the cytoplasm and subsequently. The mitochondria were stained with the mitochondria-specific Mito Tracker Red CMXRos dye as a marker in the cytoplasm (Fig. 6).

### ONC-V3 inhibited motility of HO-8910PM cells

**Fig. 7:**
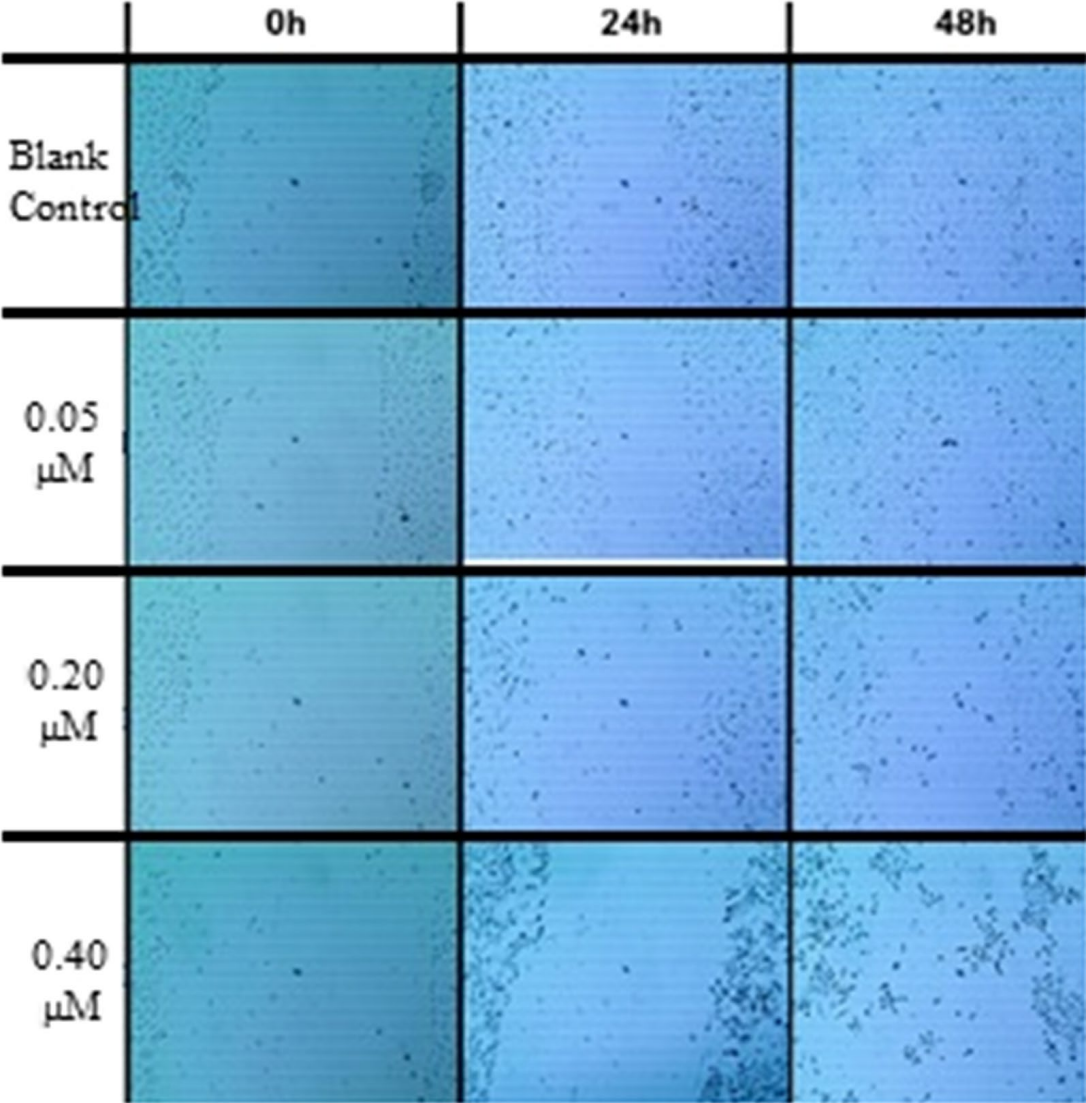
HO-8910PM cells were cultured with ONC-V3 for 48 h. The blank control group and the group of 0.05 μmol/L were basically healed. The number of neonatal tumor cells in the original scratches was slightly greater than on the scratches. The scratches in the 0.20 and 0.40 μmol/L groups were not significantly healed, indicating that high concentrations of Onc-V3 could inhibit tumor cell migration and healing. The recombinant immune toxin Onc-V3 was found to inhibit the migration ability of highly metastatic human ovarian cancer cells of strain HO-8910PM, and this ability was dose-dependent

The most essential characteristics of tumor cells are invasion and metastasis. The scratch wound-healing assay was performed to analyze whether Onconase inhibits the motility of HO-8910PM cells, in vitro. After creating a wound-like gap by scratching the cell monolayer, untreated cells were found to efficiently migrate into the gap area. However, in the presence of Onconase, HO-8910PM cells were significantly inhibited. When the cells were treated with 0.4 μmol/L ONC-V3 for 48 h, cell motility was not affected (Fig. 7), suggesting that the Onconase effect on cell migration was not due to its cytotoxicity (Table 2).

**Table 2:** Results of the wound healing experiments

	Original length (μmol/L)			Length at 48 h (μmol/L)			Healing rate (%)
Blank control	829	914	784	0	0	0	100
0.05	830	702	577	71	65	29	92.39 ± 2.27
0.10	714	815	572	94	108	78	86.65 ± 0.25^#^
0.20	928	691	825	325	221	289	65.99 ± 1.76^#^
0.40	812	658	502	490	422	316	37.52 ± 1.94^#^
0.80	901	847	956	783	749	810	13.31 ± 1.86^#^
1.60	793	933	960	740	892	923	4.98 ± 1.50^#^
3.20	947	809	716	902	758	679	5.41 ± 0.80^#^
Positive control	812	762	892	352	315	411	56.41 ± 2.38^#^

Results are expressed as mean ± SD

^#^*P *≤ 0.001

### ONC-V3 suppressed migration and invasion of HO-8910PM cells

**Fig. 8:**
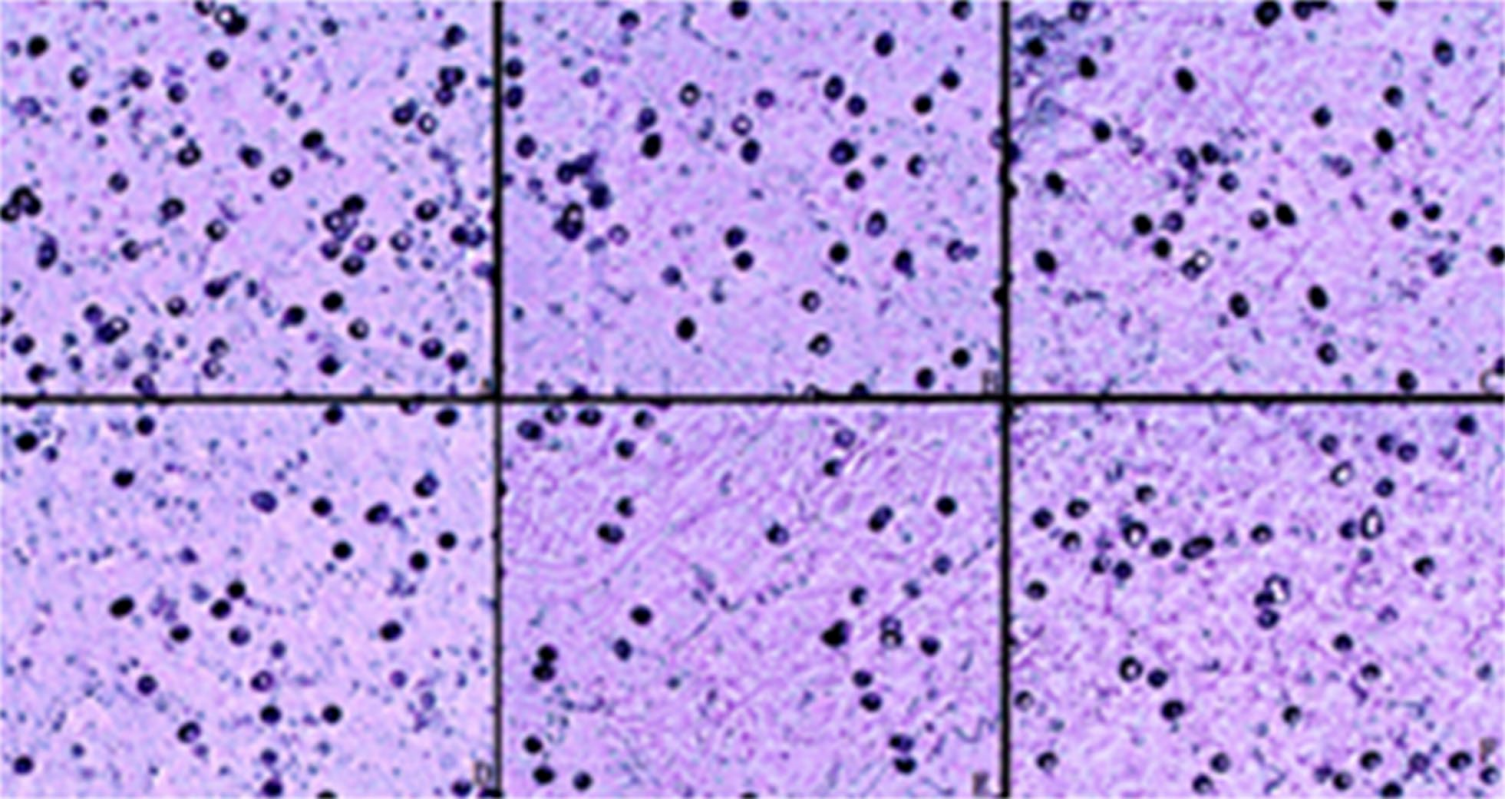
HO-8910PM cells were cultured with ONC-V3 for 48 h. Unlike in the blank control group, the number of invasive cells penetrating the basement membrane gradually decreased as the concentration increased. The results showed the recombinant immunotoxin Onc-V3 to have an inhibitory effect on the invasion of HO-8910PM cells

The transwell invasion assay demonstrated a significant decrease in the migration of ONC-V3 treated cells. These findings suggested that ONC-V3 could inhibit the migration and invasion of HO-8910PM cells (Fig. 8, Table 3).

**Table 3:** Cultivation of HO-8910PM cells with different concentrations of Onc-V3 for transwell

Onc-V3 (μmol/L)	The number of cells
Blank control	201
0.20	183
0.40	157
0.80	112
1.60	74
Positive control	168

### ONC-V3 suppressed migration and invasion of HO-8910PM cells

**Fig. 9:**
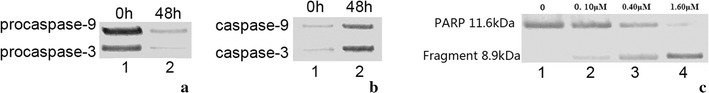
HO-8910PM cells were cultured with ONC-V3 for 48 h.** a** procaspase-9 and procaspase-3 decreased in Onc-V3-induced apoptosis.** b** caspase-9 and caspase-3 increased.** c** PARP decreased along with the dose of ONC-V3, while the fragments of cleaved PARP increased

Western blot results showed significantly decreased PARP, procaspase-9 and procaspase-3 in Onc-V3-induced apoptosis (Fig. 9a, c). And the fragment of PARP, caspase-9 and caspase-3 increased (Fig. 9b, c). These suggested that Onc-V3 may induce tumor cell apoptosis via the caspase pathway, which were consistent with other reports in the literature.

## Discussion

Biotherapy is currently the most popular mode of therapy to treat cancer. Immunotoxins are a kind of biotherapy that has made significant strides in cancer treatment [6, 7]. The ability of immunotoxins to kill cancer cells could be due to their capacity to function as antimicrotubular agents, DNA minor groove binding agents, and/or alkylating agents. Often immunotoxins are able to execute these biological activities in concentrations ranging from ng/kg to µg/kg. The high cytotoxicity is an important feature to address the safety challenge of immunotoxins for systemic application in cancer patients. The improvements in toxicity of immunotoxins have accelerated the clinical development of a new generation of immunotoxins and have resulted in the recent approval of new drugs.

Another emerging problem was immunogenicity, which was elicited by many bioengineered and expressed immunotoxins after administering to cancer patients. The immune response would obviate the metabolic half-life and impose restrictions on further drug development, thus holding back their clinical use. Therefore, efforts have been made to reduce the immune response successfully.

Onconase, an RNase A like ribonuclease found in the oocytes of the Northern leopard frog, could degrade RNA and lead to cancer cells apoptosis [8–11]. In a previous study, we chose RNase like Onconase to invent a novel immuno- RNase to target CXCR4 receptors on the surface of cancer cells. We placed ONC-V3 gene downstream of the truncated *Saccharomyces cerevisiae* α-mating factor-pre (α-MF-pre) secretion signal with the *P. pastoris* GAP promoter, and developed a preparation method. In this study, we were able to preliminarily detect its anti-cancer effect in vitro.

The CXCR4 chemokine receptor was overexpressed in more than 20 types of cancers, including breast cancer, ovarian cancer, glioma, pancreatic cancer, prostate cancer, AML, B-CLL, melanoma, cervical cancer, colon carcinoma, rhabdomyosarcoma, astrocytoma, small-cell lung carcinoma, CLL, renal cancer, and non-Hodgkin’s lymphoma [18]. Biotherapies that target CXCR4-overexpressing cancer cells may be feasible.

In this study, the results of the in vitro experiments demonstrated that the ONC-V3 conjugate improved the anti-cancer effect compared with Onconase, suggesting that V3 could help Onconase to target the cancer cells in order to significantly enhancing its cytotoxicity. Our internalization results demonstrated that most ONC-V3 was around HO-8910PM cells and that several molecules reached the cytosol after 1 h, deducing that ONC-V3 may move immediately from endosomes into the cytosol, as reported for Onconase. The Cytochrome C was bind to tRNA to avoid the formation of apoptosome, while Onconase would degrades tRNA specifically. The Cytochrome C was liberated and the apoptosome was built together with Apaf-1 along the help of ATP. Then the pre-caspase 9 joined in the apoptosome and transferred to the active form caspase 9. Caspase 3 was activated by caspase 9, so the pre-caspase 3 reduced. Along with the Cytochrome C, AIF was released from mitochondria to cytosol. It induced the degradation of nucleus, thus the PARP was cleaved and reduced. The mechanism of ONC-V3 to inhibit the cancer cells was similar to the Onconase, while it increased the target ability, in other words, the cytotoxicity.

## Conclusion

In this study, the constructed immunotoxin showed its good circulatory stability while it could induce the cancer cell apoptosis specifically. Therefore, the ONC-V3 conjugate designed in the present study could be used as a novel potential anticancer drug for further studies.

## Abbreviations

CXCR4: chemokine receptor 4
Gln: glutamine residue
Onc-V3: Onconase-(V3)_2_
RNase A: ribonuclease A
OD: optical density
FITC: fluorescein isothiocyanate
